# Physical Examination of the Hip: Assessment of Femoroacetabular Impingement, Labral Pathology, and Microinstability

**DOI:** 10.1007/s12178-022-09745-8

**Published:** 2022-02-16

**Authors:** Stephanie E. Wong, Charles J. Cogan, Alan L. Zhang

**Affiliations:** Department of Orthopaedic Surgery, University of California, San Francisco, 1500 Owens Street, San Francisco, CA 94158 USA

**Keywords:** Physical examination of the hip, Femoroacetabular impingement syndrome, Hip microinstability, hip arthroscopy, provocative maneuvers

## Abstract

**Purpose of Review:**

Determining the correct diagnosis can be challenging in patients presenting with hip pain. The physical examination is an essential tool that can aid in diagnosis of hip pathology. The purpose of this review is to provide an updated summary of recent literature on the physical exam of the hip, particularly as it relates to diagnosis of femoroacetabular impingement (FAI) syndrome, labral injury, and hip microinstability.

**Recent Findings:**

Physical exam findings consistent with the diagnosis of FAI include reduced supine hip internal rotation and positive flexion-adduction-internal rotation maneuvers. Labral tears can be detected on exam with the Scour test. Studies demonstrate altered hip biomechanics in patients with FAI during activities such as walking and squatting. Those with FAI have slower squat velocities, slower sit-to-stand tests, and increased hip flexion moments during ambulation. Hip microinstability is a dynamic process, which can occur after prior hip arthroscopy. For hip microinstability, the combination of the three following positive tests (anterior apprehension, abduction-extension-external rotation, and prone external rotation) is associated with a 95% likelihood of microinstability as confirmed by examination under anesthesia at the time of surgery.

**Summary:**

A comprehensive hip physical exam involves evaluation of the hip in multiple positions and assessing hip range of motion, strength, as well as performing provocative testing. A combination of physical exam maneuvers is necessary to accurately diagnose FAI syndrome and labral pathology as individual tests vary in their sensitivity and specificity. While an elevated level of suspicion is needed to diagnose hip microinstability, the provocative tests for microinstability are highly specific.

## Introduction

While a multitude of pathologies can affect the hip and surrounding structures, physical examination remains one of the most valuable tools physicians can utilize to diagnose disease. Advances in our understanding of hip anatomy, biomechanics, and physiology have led to a variety of hip-specific maneuvers that have enhanced the clinician’s examination. This paper reviews the approach to a hip physical exam that includes upright and supine exam topics as well as provocative maneuvers and a discussion on the physical exam for microinstability of the hip.

## Upright Exam

Upright examination of the patient can provide crucial information for detection of hip pathology and includes evaluation of both stance and gait. While standing, one can observe the posture of the hip as well as the adjacent joints, specifically the lumbar spine and knee. Flattening of the lumbar spine or excessive lumbar lordosis should be noted. A slightly flexed position of the involved hip and ipsilateral knee may indicate hip joint irritability [1]. Depending on the etiology of the pain, the patient may hold the hip flexed to protect against weight-bearing and loading of the hip (such as in the case of a femoral neck stress reaction). The position of hip flexion also maximizes the intracapsular volume of the hip joint, which can be seen in pathologies such as septic hip.

**Figure 1:**
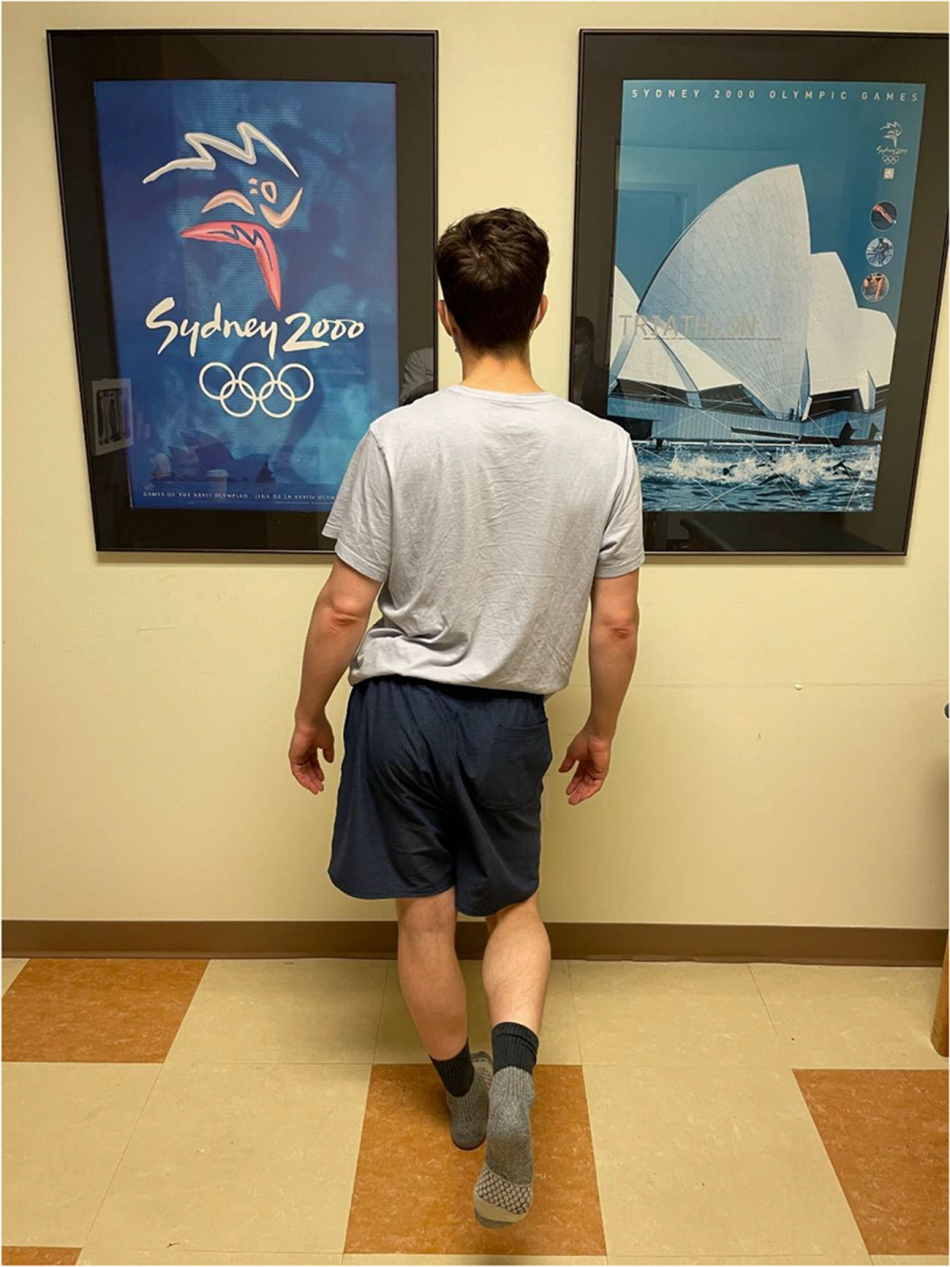
Trendelenburg sign

Single-leg stance requires engagement of the hip abductors and can detect pathology such as gluteal tendon tears (gluteus medius and/or minimus). A positive Trendelenburg sign occurs when a patient performs a single-leg stance and the contralateral pelvis drops due to abductor weakness [2]. Frequently, the torso then compensates by leaning toward the side of the pathology. The authors perform this exam while seated behind the patient, palpating the posterosuperior iliac spine with each thumb, and placing the hands around the iliac crest. Then the patient is asked to lift one foot off the ground for at least 30 s. There is a positive Trendelenburg sign if the contralateral hemipelvis drops lower than the pelvis on the stance side (Figure 1). The test is normal if the pelvis remains level [3].

The Trendelenburg gait was first described by a German surgeon, Friedrich Trendelenburg, in 1985. The Trendelenburg gait is an abnormal gait pattern due to weakness in hip abductors, primarily the gluteus medius and gluteus minimus [2], and this can be seen in cases of developmental dysplasia of the hip, chronic hip dislocations, Perthes disease, gluteal tendon tears, or other causes of hip abductor insufficiency. A Trendelenburg gait can often be correlated with the Trendelenburg sign and occurs with the pelvis dropping on the opposite side of the stance leg. A patient with bilateral pathology will have a waddling-type gait, where the pelvis drops on the contralateral side with each step.

An antalgic gait should be distinguished from a Trendelenburg gait. An antalgic gait occurs with shortening of the stance phase, increased hip flexion, and avoidance of hip extension on the affected side [1]. Of note, the pelvis remains level with an antalgic gait. It is worthwhile to have all patients walk down the clinic hallway in order to properly assess for subtle asymmetries in their gait and evaluate their coronal and sagittal balance. Foot progression angle can also be assessed and can be correlated with femoral version [4]. In-toeing or an internal foot progression angle can be associated with increased femoral anteversion, while out-toeing or an external foot progression angle can be associated with femoral retroversion. In-toeing can also occur with internal tibial torsion, in contrast to out-toeing which can be due to external tibial torsion, so it is important to assess for any contribution from the level of the tibia.

Hip biomechanics are altered in patients with femoroacetabular impingement (FAI), particularly during walking, squatting, and climbing stairs [5–9]. For patients that are able to ambulate unassisted, a double-leg squat can be indicative of a patient’s dynamic strength and balance. Asking the patient to perform a double-leg squat without much additional prompting can reveal much about their posture, form, and stability. Take note of the depth of the squat, valgus collapse at the knee joint, or any coronal plane truncal shifts. A recent biomechanical study demonstrated that patients with FAI had slower squat velocities during both ascent and descent for a double-leg squat preoperatively compared to controls [10]. Studies by Samaan et al. showed that patients with FAI had increased hip flexion loading moments compared to controls and shortened hip extension phase with walking [6, 11].

**Figure 2:**
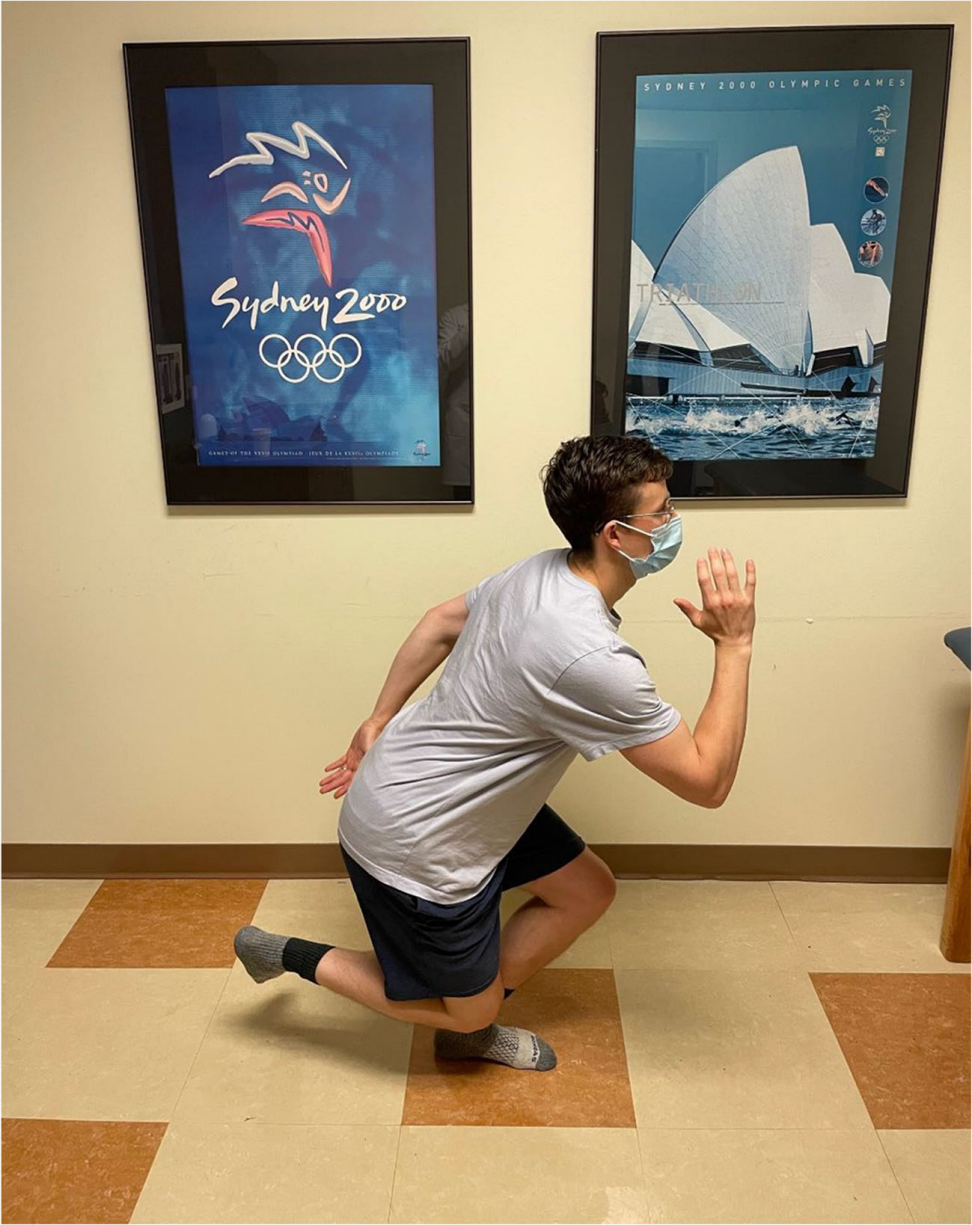
Single-leg squat

The single-leg squat is much more challenging to perform compared to the double-leg squat (Figure 2). For those patients who can perform a double-leg squat with ease, a single-leg squat can reveal side-to-side differences. A study comparing hip biomechanics during double and single-leg squats showed exaggerated biomechanical differences during the single-leg squat task in patients with FAI, with slower squat velocities, less peak hip adduction, and lower hip abduction and extension moments [5]. Similar to the double-leg squat, one should assess the squat in both the coronal and sagittal plane, paying attention to form, stability, and depth.

## Supine Exam

The supine exam can take place either before or after the upright exam. The authors prefer to begin the supine exam with a log roll test, which has been described as one of the most specific tests for hip pathology [1]. The limb is gently rolled back and forth, moving the femoral head within the acetabulum, and is positive if the patient has pain, which indicates intra-articular pathology. It is preferred to perform this test with the examiner’s hand on the thigh to avoid confounding this test with any knee pathology. This test avoids significant force or excursion on adjacent muscles and, therefore, is an appropriate initial test for any patient with hip pain.

**Figure 3:**
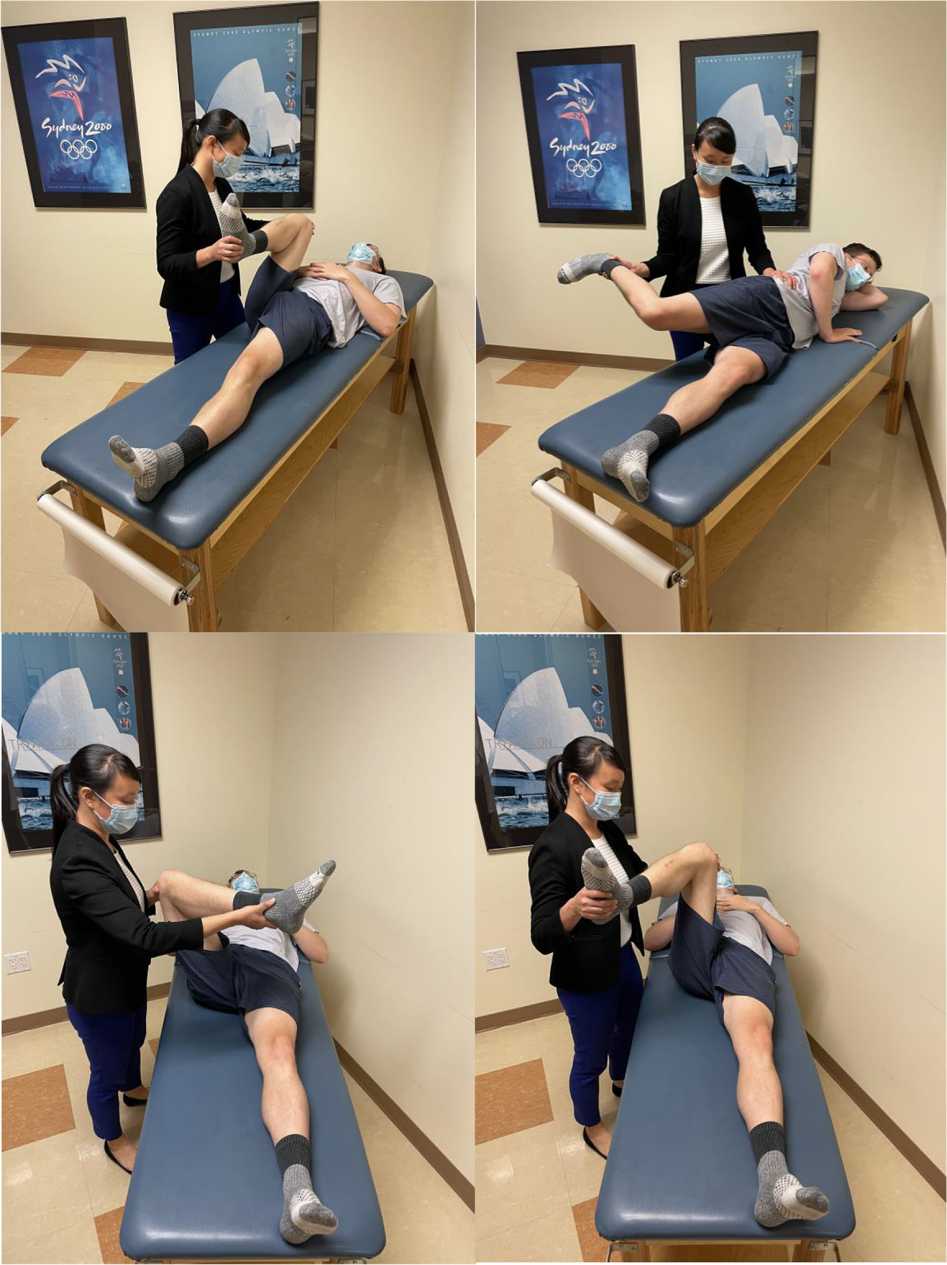
Hip range of motion. Top left shows hip flexion. Top right shows hip extension. Bottom left shows hip external rotation. Bottom right shows hip internal rotation

Hip range of motion is then assessed (Figure 3). Passive hip flexion involves the examiner flexing the hip and knee toward the patient’s chest and measuring the passive hip flexion obtained, with normal hip flexion approximately 120°. However, impingement-free hip flexion is likely less than this. A dynamic ultrasound study investigating hip motion in asymptomatic, young adult females showed that with passive hip flexion, initial labral deflection occurred at 72°, and bony impingement occurred at 101° [12]. It is important to note that rotation of the hip is directly affected by version of the proximal femur. Hip internal and external rotation can be measured in the prone or supine position. Hip rotation measurements are similar in both supine and prone positions with good inter-observer reproducibility [13]. Hip internal and external rotations are then assessed in the supine position with the hip and knee flexed to 90°. A study on asymptomatic volunteers demonstrated excellent inter-rater reliability for hip flexion (ICC 0.87) and supine hip internal rotation (ICC 0.75) [14]. Supine external rotation (ICC 0.18) and supine hip abduction (ICC 0.34) were found to be the least reliable measurements [14]. Obligate external rotation occurs when hip external rotation accompanies passive hip flexion and can indicate intra-articular hip irritation and is seen in conditions such as slipped capital femoral epiphysis, femoroacetabular impingement, and hip arthritis. Decreased hip internal rotation can be present in FAI and has been associated with progressive osteoarthritic changes on imaging in young athletes with hip internal rotation less than 10° [15]. Hip extension is best assessed with the patient lying in the lateral decubitus position. The examiner passively extends the top limb with the knee flexed to 90°, measuring hip extension which is typically 5 to 10°.

Palpation is generally unrevealing for patients with FAI but may help identify other causes of pain including those from the spine, lateral hip, and pubic symphysis [1]. The structures that can be palpated in the supine position include the hip flexor muscle group anteriorly. Tenderness over the hip flexors can indicate hip flexor injury, or irritation/tendonitis as a cause of the patient’s groin pain, in contrast to intra-articular causes of groin pain, which are typically not palpable. The bony landmarks that can be palpated include the pubic symphysis (tender with osteitis pubis and with core muscle injuries), the greater trochanter (tender with trochanteric bursitis), and the anterior superior iliac spine (ASIS). The lateral hip structures can also be palpated from the supine position, and they include the trochanteric bursa and iliotibial band.

Muscle strength testing is then performed and rated on the manual muscle testing scale out of five, with a score of five indicating full strength against resistance. Hip flexion strength can be assessed in the supine position, asking the patient to raise their leg in the air with the knee straight. Resistance testing can also be performed with the examiner’s hand pushing down on the ankle while the knee is straight and hip flexed to 30° and asking the patient to actively resist. This test for hip flexion strength is similar to the Stinchfield test which is discussed later in this chapter. Hip abduction and adduction can be tested in the supine position with the knees flexed to 90° with feet planted flat on the exam table. To test hip abduction, the examiner places their hands on each side of the patient’s knees, and the patient is asked to push outwards. To test hip adduction, the examiner moves their hands in between the patient’s knees, and the patient is instructed to pull inwards.

## Provocative Maneuvers

A variety of provocative maneuvers are important for the complete evaluation of both intra- and extra-articular hip pathology. It is important to note that there is wide clinical variety in the description and application of these various maneuvers, and there is minimal information on the diagnostic validity and accuracy of these tests for the diagnosis of FAI [16]. As such, the Warwick international consensus statement in 2016 called for a combination of the proper symptoms, positive clinical signs, and imaging findings in order to diagnose FAI syndrome [17].

## Femoroacetabular Impingement and Labral Pathology Tests

### Flexion-Adduction-Internal Rotation (FADDIR)

**Figure 4:**
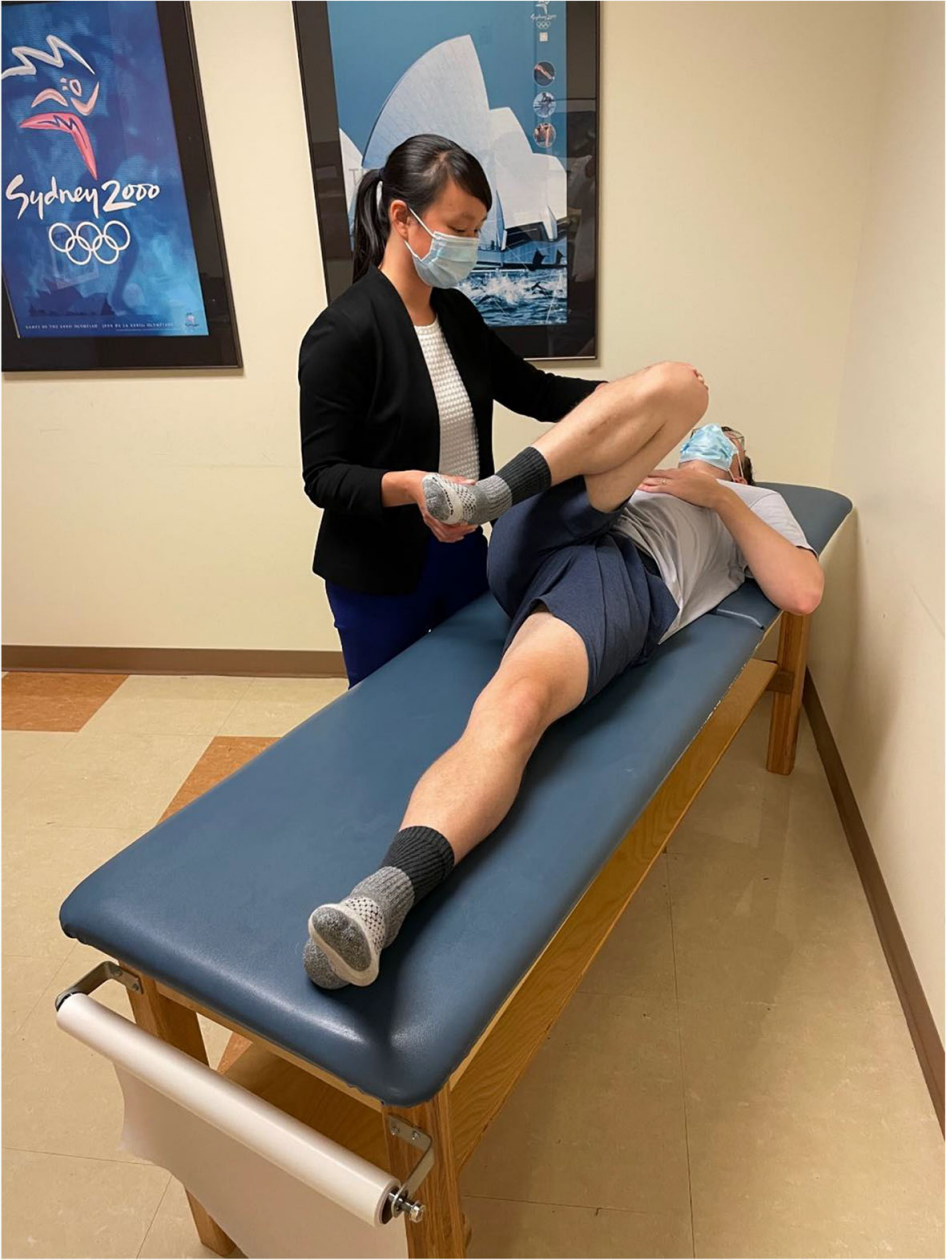
Flexion-adduction-internal rotation (FADDIR)

This provocative maneuver is used to assess presence of FAI and labral pathology (Table 1). This phenomenon was first noted by Reinhold Ganz in early descriptions of FAI [18]. The test has been described in both the supine and lateral recumbent position (with the affected side up). The examiner passively brings the hip into flexion, adduction, and internal rotation (Figure 4). Reproduction of pain is considered a positive test and diagnostic of FAI [16, 19, 20]. One study of patients with symptomatic hip pain evaluated the diagnostic accuracy of the anterior impingement test and FADDIR test, which they found to be 80%; however, their specificity was quite low at 26% and 24%, respectively [21]. Often a combination of physical exam maneuvers throughout a range of motion is the best way to dynamically stress the hip on exam to potentially reproduce the symptoms causing discomfort, and this was reflected in their same study, as the most specific maneuver was passive hip range of motion in an internally rotated and neutrally flexed hip [21]. When testing FADDIR specifically in 90° of hip flexion, this maneuver is sometimes referred to as the anterior impingement test [16, 22]. Sensitivity of anterior impingement test to detect FAI ranges from 60 to 100% and inter-observer agreement is excellent at 96% [14, 16, 23, 24].

**Table 1:** Exam maneuver sensitivity and specificity

Test	Sensitivity	Specificity
Flexion-adduction-internal rotation [10]	59–100%	10–100%
Scour Test [10, 19]	50–100%	29%
Internal rotation over pressure [10, 20]	75–89%	15–43%
Posterior impingement test [22]	21%	N/A
Flexion-abduction-external rotation [10]	41–97%	18–100%
Resisted straight leg raise	6–75%	38–100%
Thomas [26]	89%	92%
Anterior apprehension test [32]	71%	85%
Abduction-extension-external rotation [32]	81%	89%
Prone external rotation [32]	33%	98%

### Scour Test (Labral Stress Test)

The scour test is a dynamic maneuver designed to assess labral pathology. The hip is brought from a flexed and internally rotated position into hip abduction and extension while axial compression is applied. This maneuver is likely to elicit pain or mechanical symptoms in the setting of an anterior labral tear, as bony impingement of the labrum reproduces symptoms. The application and interpretation of this maneuver is analogous to a McMurray Test in the knee. Sensitivity of the scour test has ranged in the literature from 50 to 100% [16, 25]. It is important to note the degree of flexion at which symptoms are reproduced, as this may give clues about the position of anterior labral pathology.

### Internal Rotation Over Pressure (IROP)

This maneuver is used to assess both FAI and labral pathology. The patient lies in the supine position, and the affected leg is brought into flexion and internal rotation with the application of axial compression. Reproduction of pain is considered a positive test. Sensitivity of this maneuver has been reported to be 75–89% [25, 26]. Maslowski et al. compared IROP, Scour test, FABER, and Stinchfield tests, and they found IROP to have the highest positive predictive value for intra-articular hip pathology [25].

### Posterior Impingement Test

**Figure 5:**
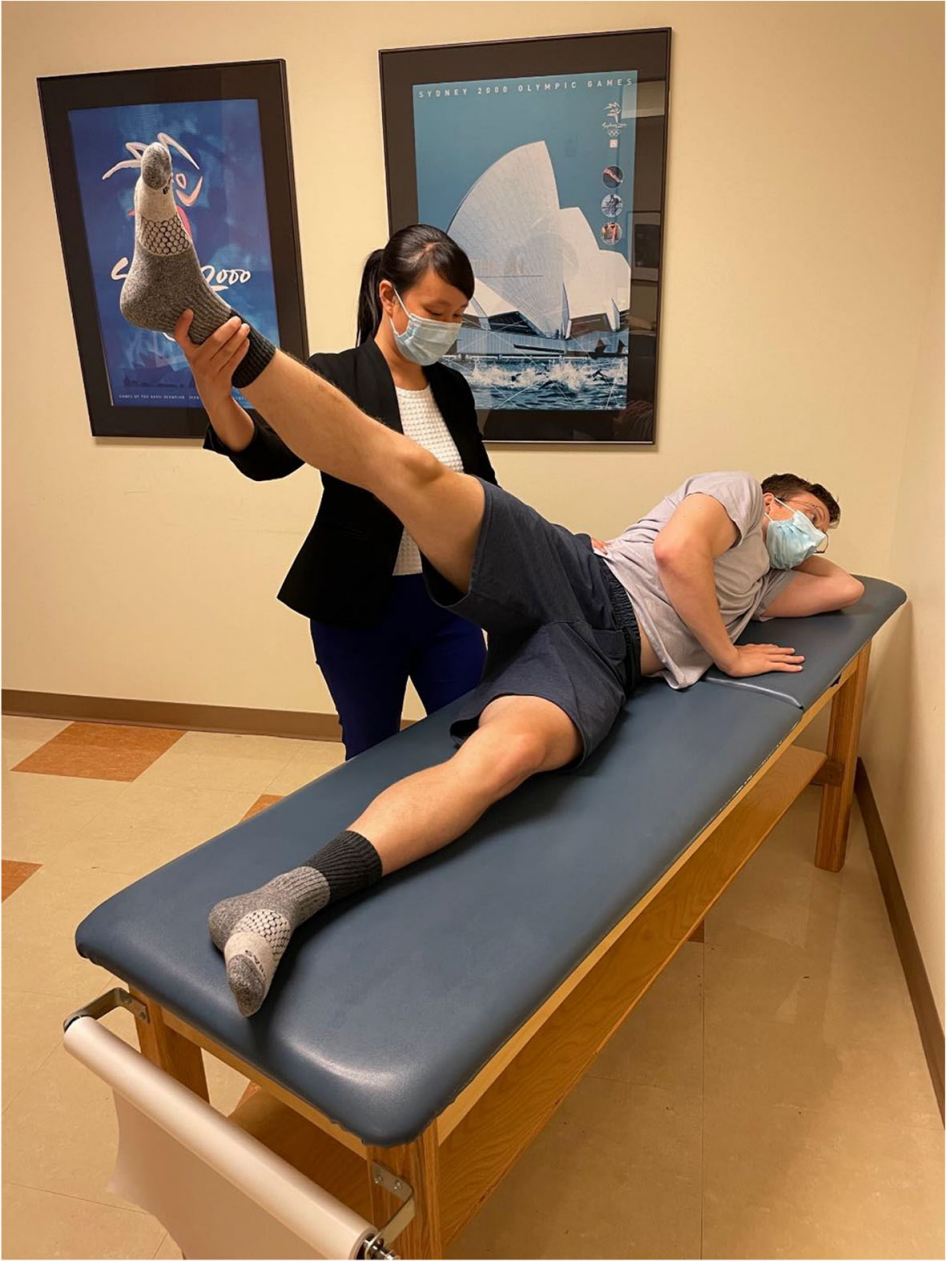
Posterior impingement test

This maneuver is used to assess labral impingement in the posterior hip, often a result of global acetabular overcoverage. This test is performed with the hip in full extension, maximal abduction, and external rotation (Figure 5). The test is positive when the patient describes posterior hip pain [27]. Sensitivity for this exam has been reported at 21% [28], which is exemplary of the fact that posterior hip impingement is difficult to diagnose on physical exam. Patients often present with vague lateral hip, buttock, thigh, or lumbar spine pain, and diagnosis is reliant upon a combination of physical exam maneuvers and MRI [29].

### Flexion-Abduction-External Rotation (FABER/Patrick Sign)

**Figure 6:**
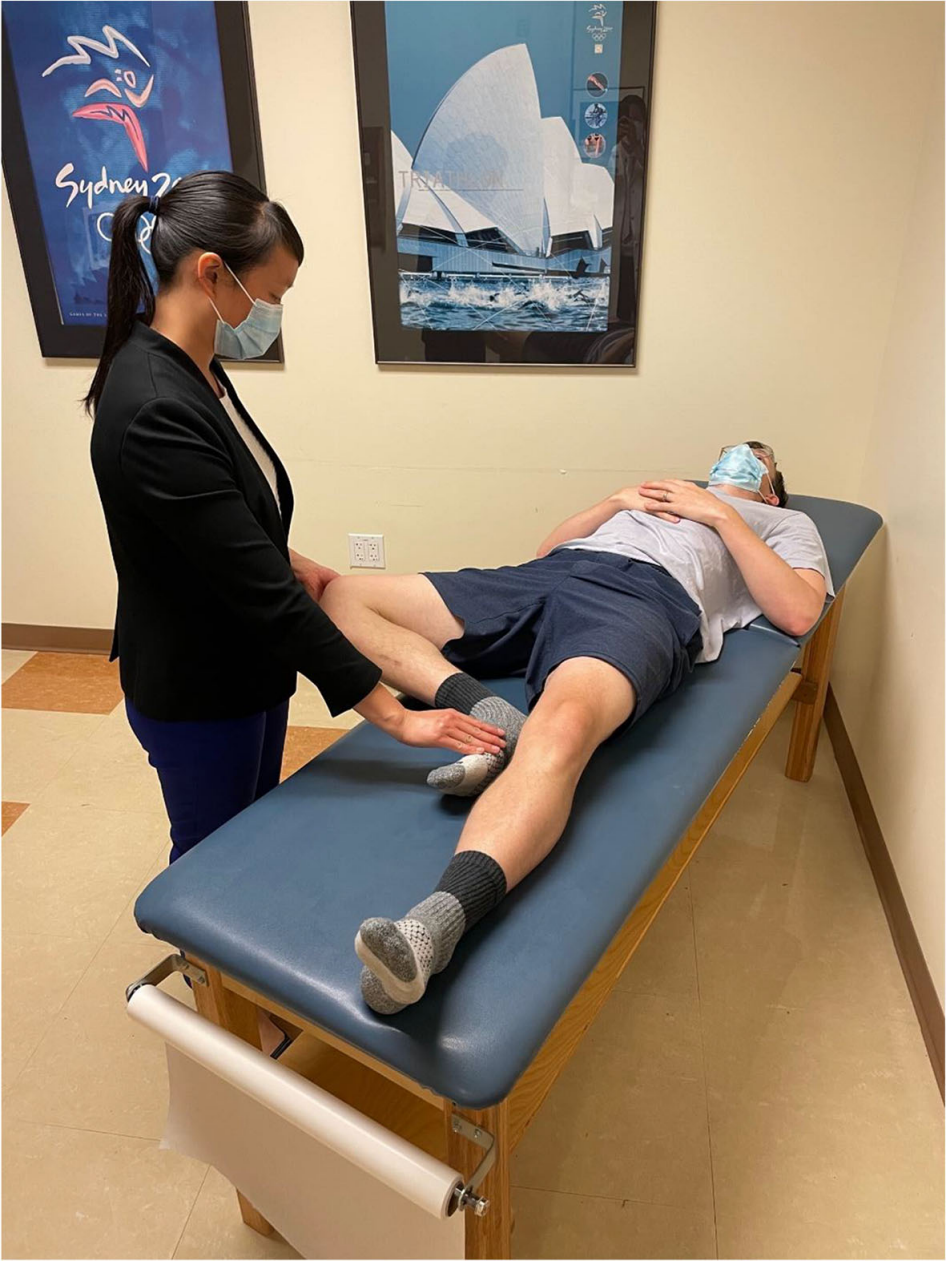
Flexion-abduction-external rotation (FABER)

This maneuver is used to assess both FAI/labral pathology as well as sacroiliac (SI) joint pathology. The test is performed with the patient in the supine position. The affected hip is simultaneously flexed, abducted, and externally rotated, so that the ankle rests just proximal to the contralateral knee (Figure 6). With the affected limb ASIS stabilized, the knee is pressed toward the floor. A positive test is remarked as either decreased range of motion compared to the contralateral hip or reproduction of pain. Systematic review of diagnostic accuracy for physical exam maneuvers of the hip demonstrated sensitivity of FABER ranging from 41 to 97% and specificity from 18 to 100% [16], and another study of asymptomatic volunteers demonstrated 98% inter-observer agreement for this maneuver [14]. It is important to carefully illicit location of pain with this maneuver, as posterior pain on the contralateral side may be more indicative of SI joint pain, whereas ipsilateral anterior hip or groin pain may represent intra-articular pathology, such as labral tear [30].

## Other/Extra-Articular Maneuvers

### Resisted Straight Leg Raise (Stinchfield Test)

**Figure 7:**
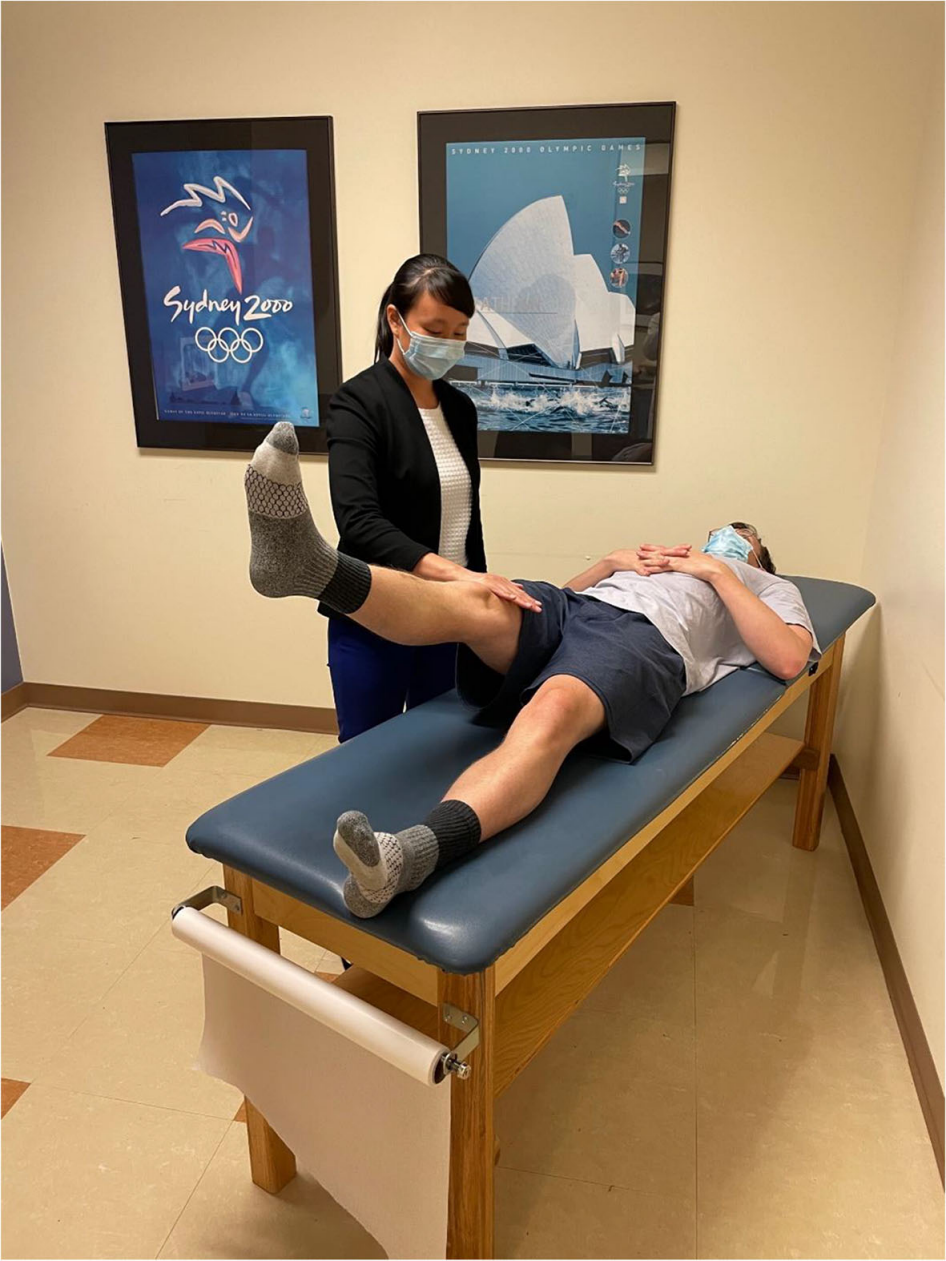
Resisted straight leg raise (Stinchfield test)

This maneuver is used to assess hip flexor/psoas inhibition due to direct tendinosis or capsulitis as the psoas compresses the hip capsule during resisted hip flexion [30]. The test is performed in the supine position. The patient is asked to flex their hip against resistance at 45° of hip flexion with the knee extended (Figure 7). The reproduction of pain or significant weakness with this maneuver constitutes a positive test. The sensitivity of this maneuver for diagnosis of FAI or labral pathology is quite limited with a range from 6 to 75% in the literature [24, 25, 28]. Sensitivity and specificity for the Stinchfield test is also limited for evaluation of psoas pathology at 62% and 25%, respectively [31] This non-specific maneuver is used to evaluate many hip pathologies, including degenerative pathologies and trauma, and the literature has demonstrated specificity ranging from 38 to 100% [16].

### Thomas Test

**Figure 8:**
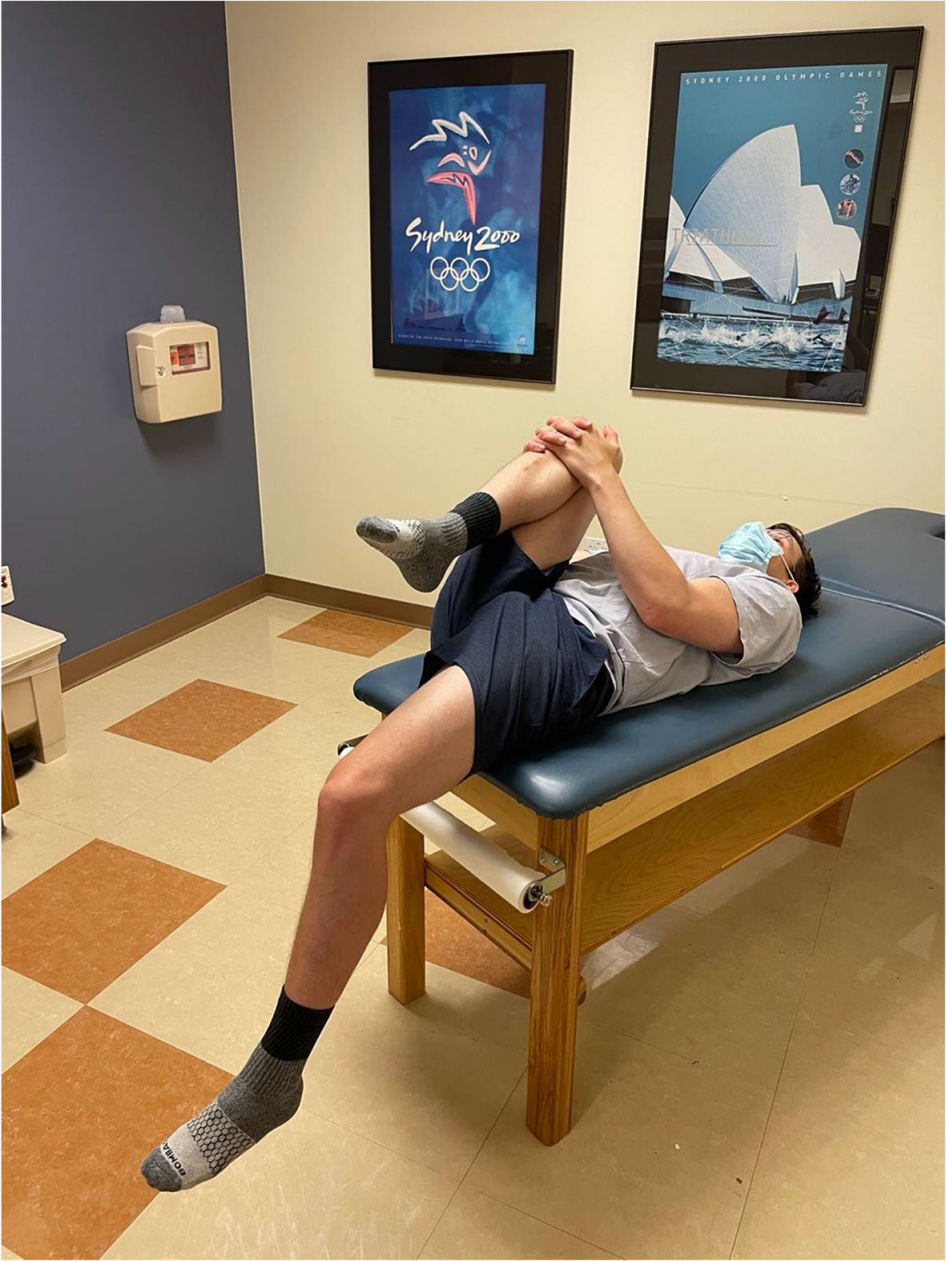
Thomas test

This maneuver is classically used to assess for hip flexion tightness or contracture; however, it has also been described to help in diagnosis of labral pathology. In this test, both of the patient’s hips are fully flexed in the supine position. With the contralateral hip held in flexion, the affected hip is extended off the edge of the table fully (Figure 8). The test is considered positive for labral pathology when a click is palpated or pain is elicited [26]. Additionally, the test is positive for a hip flexion tightness or contracture when the affected hip cannot be extended back to neutral. Sensitivity of the Thomas test for diagnosis of a labral tear has been described as 89% and specificity of 92% [32]. Hip tightness can also be assessed in the lateral position with the Ober test, where the hip is slowly adducted from an extended and abducted position until motion is restricted. This test assesses tightness of the iliotibial band along the lateral thigh [1].

One can also evaluate for coxa saltans interna (internal snapping hip) by alternating from a FABER position to an extended, adducted, and internally rotated position. This active range of motion can elicit a snapping sensation as the iliopsoas tendon snaps over the iliopectineal eminence or the femoral head [33]. Patients often describe this sensation as a snapping with a clicking or catching sensation over the anterior hip. Additionally, one can evaluate for iliotibial band snapping over the greater trochanter with active flexion of the hip followed by passive extension and abduction in the lateral position Alternatively, one can do the hula-hoop test, where the patient is in a standing position and adducts the hip with circumduction, which can recreate snapping of the iliotibial band over the greater trochanter [34].

### Gear Stick Sign

This maneuver is used to diagnose pain related to greater trochanteric impingement, which is a less commonly encountered source of retro-trochanteric hip pain surmised to occur when the greater trochanter impinges on the ischium. The test is performed with the patient in the lateral position, with the affected side up. With the knee and hip in extension, the hip is passively abducted by the examiner. The test is positive if the maneuver elicits pain in the lateral hip at the position of maximally tolerated abduction [30]. Other tests can also be used to simulate greater trochanteric impingement. One anatomic study of 23 skeletonized cadavers noted impingement with the FABER position in 96% of specimens and 100% intra- and inter-rater reliability [35]

## Microinstability

Microinstability of the hip is defined as supraphysiologic hip motion that causes pain or discomfort with or without subjective unsteadiness of the joint, and it is believed to be caused by soft tissue injury or loss and/or bony deficiency related to developmental dysplasia of the hip, connective tissue disorders, trauma, idiopathic causes, and iatrogenic causes [36]. Another group of patients prone to microinstability is those with borderline dysplasia who may have labral hypertrophy[37]. One of the most pertinent iatrogenic causes to consider is after hip arthroscopy, as patients may have a deficient hip capsule, leading to increased motion [38]. Physical exam is key to the understanding of this process, as microinstability is a dynamic process, which is not easily diagnosed on static imaging modalities. Ultrasound imaging of the hip has shown promise in its ability to reliably and affordably assess microinstability of the hip [39, 40]

A physical exam for a patient with suspected microinstability should begin no differently than any other examination of the hip, which includes all of the previously discussed topics. Particular focus should be given to excessive range of motion (>60° in either internal or external rotation) and ligamentous laxity, as tested by Beighton’s signs [41]. The Beighton scoring system assesses joint hypermobility on a 9-point scale: 1 point for each passive hyperextension of the small finger metacarpophalangeal joint past 90°, 1 point for each thumb passive apposition to volar forearm, 1 point for each elbow hyperextending beyond 10°, 1 point for each knee hyperextending past 10°, and 1 point for forward flexion of the trunk with the knees fully extended if palms are able to touch the floor. There is no universal agreement for a cutoff value for the Beighton score. Scoring cutoffs vary and are often described as greater than 5 or 6 out of 9 points being consistent with joint hypermobility [42]. In addition to a standard exam of the hip, there are a number of provocative maneuvers that assess apprehension, range of motion, and joint stability.

### Anterior Apprehension Test (Hyperextension, External Rotation Test)

**Figure 9:**
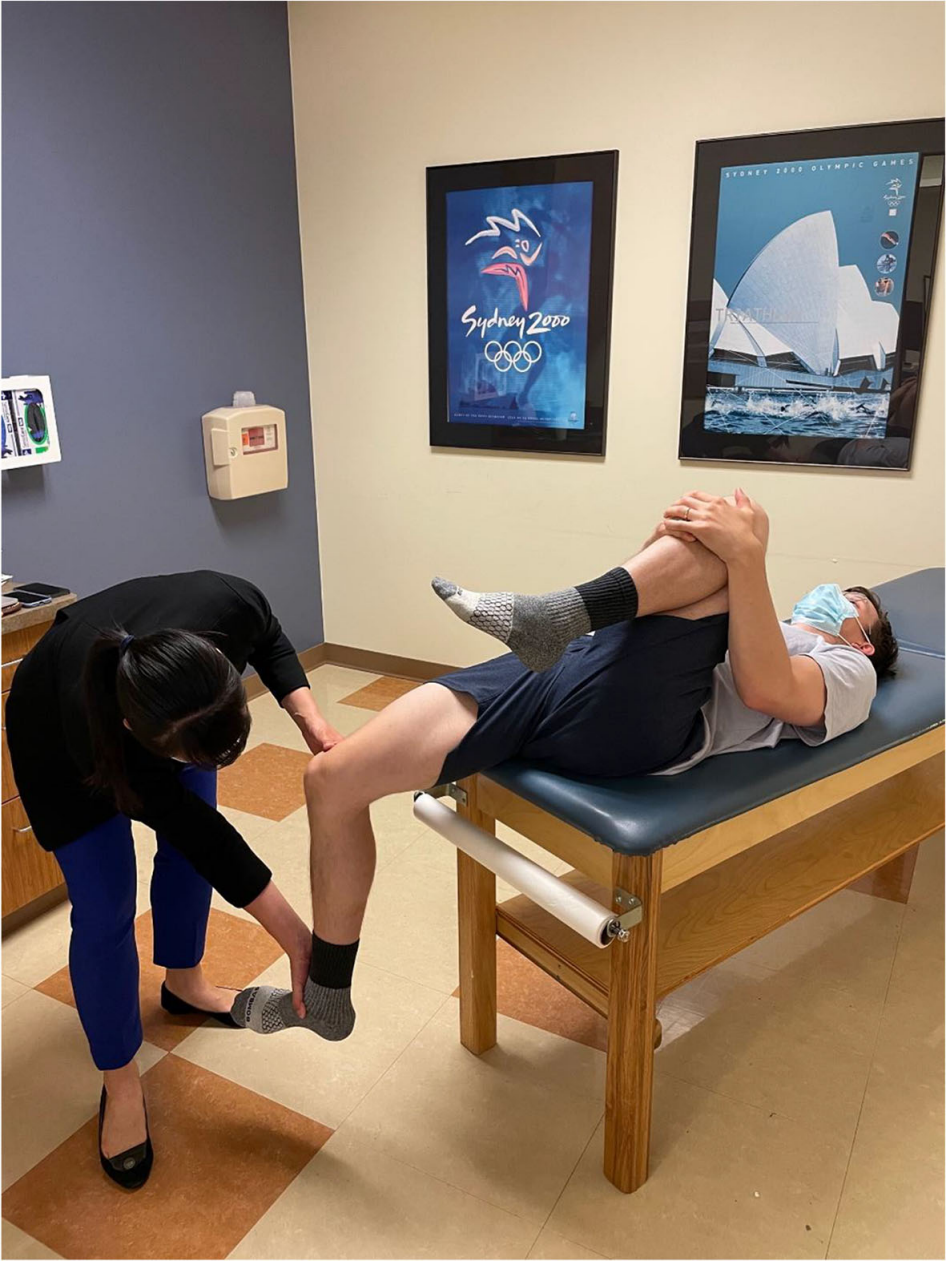
Anterior apprehension test

This test is performed with the patient supine at the end of the exam table. With the contralateral leg flexed toward the chest and the affected leg hyperextended over the end of the table, the affected hip is externally rotated, which puts the anterior capsule on stretch (Figure 9). The test is positive if this maneuver elicits anterior hip pain or apprehension [43]. Of note, posterior hip pain in this maneuver may signify posterior impingement. This test has been found to be 71% sensitive and 85% specific for the diagnosis of microinstability, with a positive predictive value of 86% [44].

### Abduction-Extension-External Rotation

**Figure 10:**
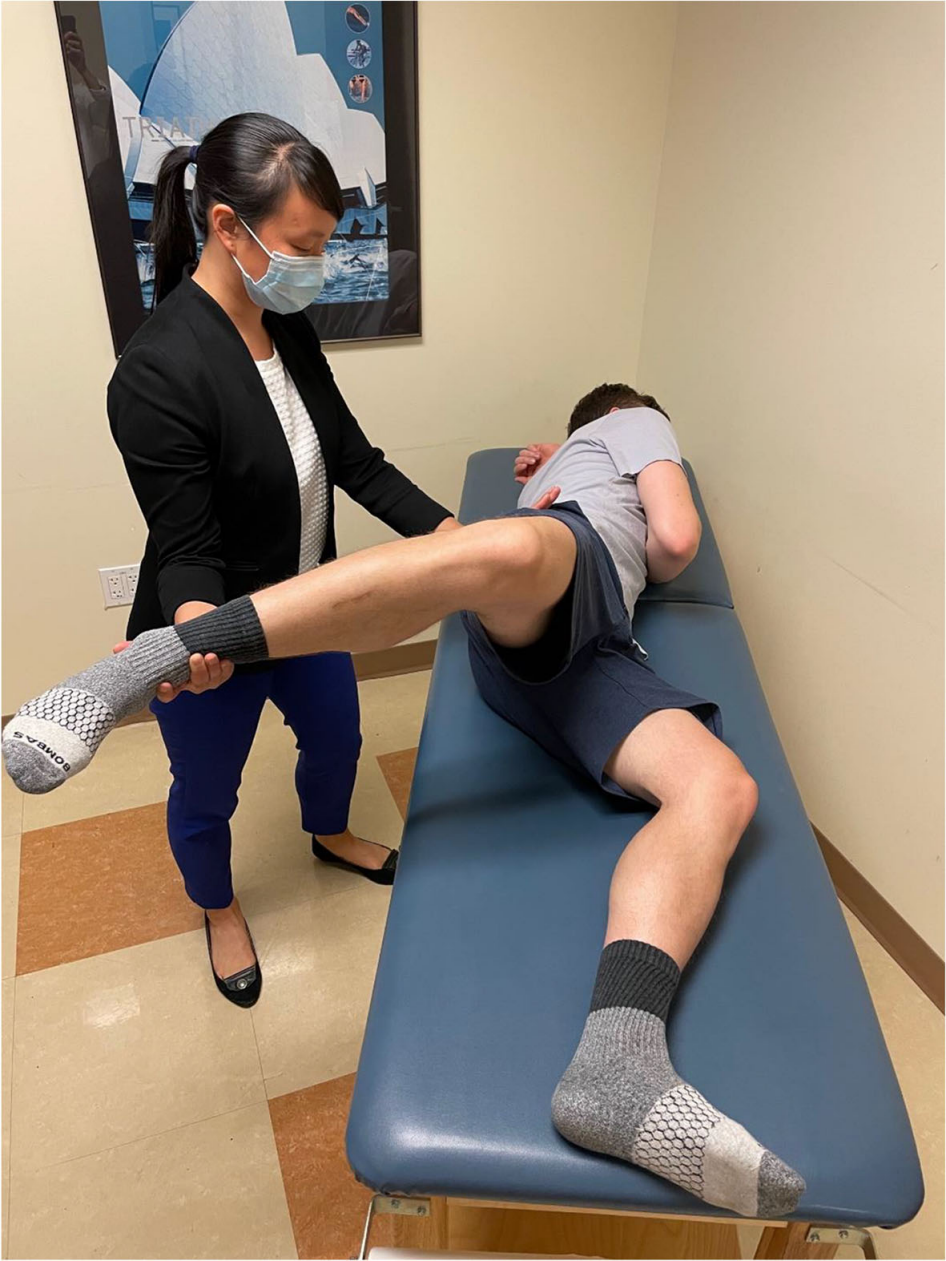
Abduction-extension-external rotation test

This test is performed with the patient in the lateral decubitus position with the affected hip up. The hip is extended, abducted to 30°, and externally rotated. The examiner then places an anteriorly directed force posterior to the greater trochanter (Figure 10). The test is positive if the patient’s symptoms of pain or apprehension are reproduced [43]. This test has been found to be 81% sensitive and 89% specific for the diagnosis of microinstability, with a positive predictive value of 91% [44].

### Prone External Rotation

**Figure 11:**
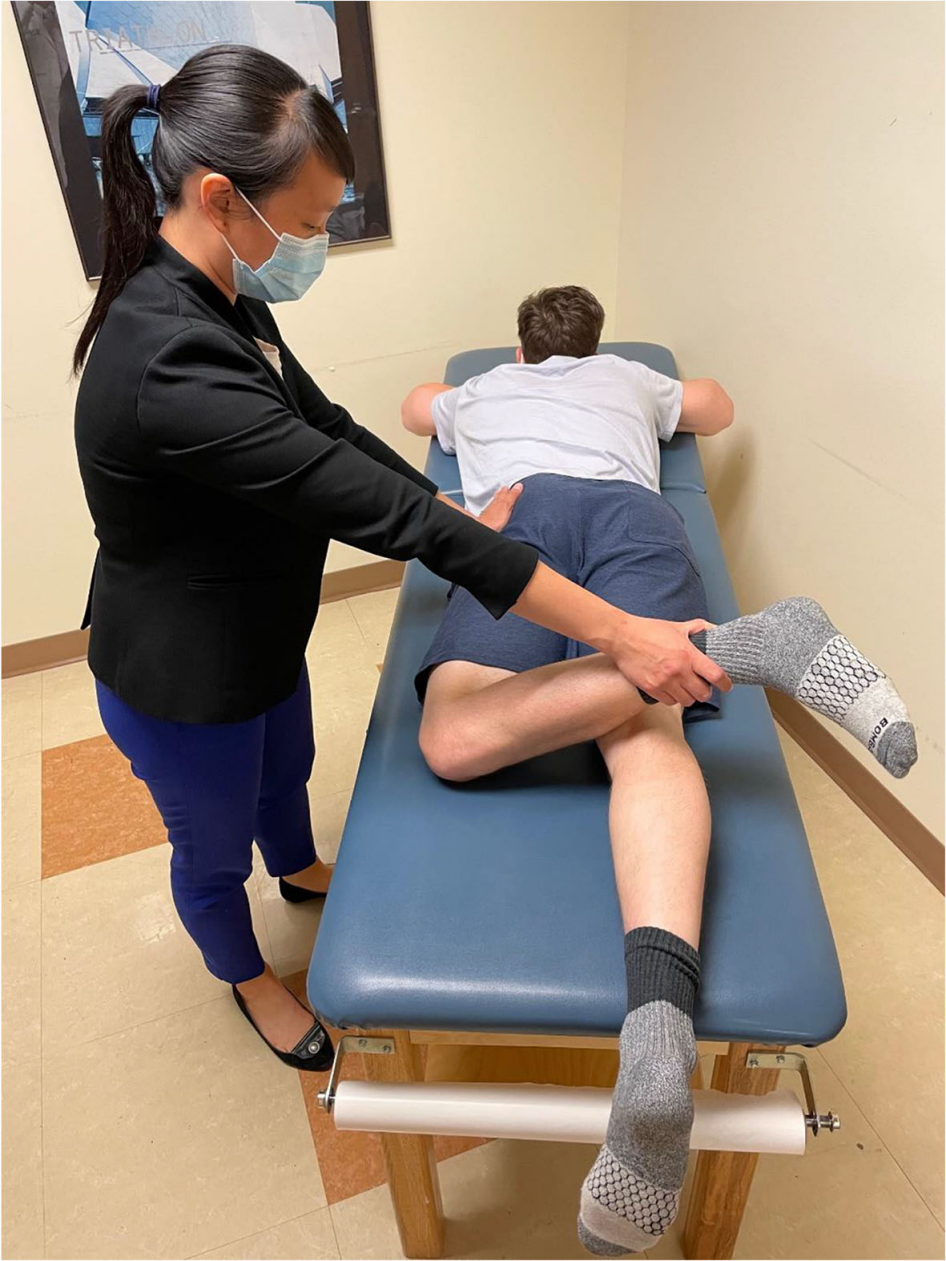
Prone external rotation test

This test is performed with the patient in the prone position and the hip in neutral flexion extension. The hip is then externally rotated and the examiner places an anteriorly directed force posterior to the greater trochanter (Figure 11). The test is positive if the patient’s pain or apprehension is reproduced [43]. This test has been found to be 33% sensitive and 98% specific. It is important to examine the patient with a number of different tests to improve diagnostic accuracy. Hoppe et al. demonstrated a 95% positive predictive value for diagnosis of microinstability if the anterior apprehension test, abduction-extension-external rotation test, and prone external rotation test are all positive [44].

### Log Roll Test (Dial Test)

This test is performed with the patient in the supine position and the knees fully extended. The examiner internally rotates the hips by turning the feet, and then the examiner removes their hands from the patient—allowing the hips to passively externally rotate. This test is positive if the angle between the foot and table in the axial plane is less than 20° [43]. It is important during this test to take note of the patient’s native femoral neck version, as decreased anteversion will lead to increased resting external rotation [45].

### Posterior Apprehension Test

While anterior hip instability is the more commonly encountered phenomenon, particularly in the setting of iatrogenic microinstability, it is important to also consider posterior hip instability, especially in the cases of prior trauma [46]. The posterior apprehension test is performed with the patient in the supine position and the affected hip flexed to 90°, adducted, and internally rotated. The examiner then applies a posterior-directed force from the knee toward the hip. The test is positive with the sensation of posterior pain or apprehension [43].

### Axial Distraction Test

This test is performed with the patient supine on the exam table. The examiner first places their knee at the ischium of the affected hip in order to help maintain stability of the pelvis during the test. Next, with the hip and knee flexed to roughly 30°, an axial distraction force is applied to the patient’s hip. The test is positive if the examiner feels a toggle or if the maneuver elicits apprehension or pain from the patient [43, 47]. This specialty test has not yet been evaluated for diagnostic accuracy.

## Conclusion

As our understanding of hip biomechanics, physiology, and pathology continues to evolve, the physical exam remains the keystone to accurate diagnosis of disease. Due to the varying sensitivity and specificity of exam maneuvers, their specific application and interpretation in the context of patient history is important. As microinstability has become better understood, physical exam maneuvers with high specificity have been developed to examine and diagnosis this pathology.
